# Conformational Dynamics of DNA Polymerases Revealed at the Single-Molecule Level

**DOI:** 10.3389/fmolb.2022.826593

**Published:** 2022-02-25

**Authors:** David P. Millar

**Affiliations:** Department of Integrative Structural and Computational Biology, The Scripps Research Institute, La Jolla, CA, United States

**Keywords:** DNA polymerase, single-molecule FRET, conformational dynamics, DNA replication fidelity, DNA-protein interactions

## Abstract

DNA polymerases are intrinsically dynamic macromolecular machines. The purpose of this review is to describe the single-molecule Förster resonance energy transfer (smFRET) methods that are used to probe the conformational dynamics of DNA polymerases, focusing on *E. coli* DNA polymerase I. The studies reviewed here reveal the conformational dynamics underpinning the nucleotide selection, proofreading and 5′ nuclease activities of Pol I. Moreover, the mechanisms revealed for Pol I are likely employed across the DNA polymerase family. smFRET methods have also been used to examine other aspects of DNA polymerase activity.

## Introduction

DNA polymerases are intrinsically dynamic macromolecular machines. During elongation of a nascent DNA strand, a polymerase must select the correct nucleotide substrate, adopt a conformation that promotes catalysis of the phosphoryl transfer reaction, release the pyrophosphate by product and move to the next templating position. This sequence of events likely involves dynamic conformational changes of the polymerase-DNA complex. In addition, many DNA polymerases harbor additional enzymatic activities, such as 3′-5′ exonuclease activity used for proofreading or 5′ nuclease activity used to remove DNA 5′ flaps. The various activities of DNA polymerases must be carefully coordinated to ensure efficient and accurate product formation. Since the respective active sites are widely separated in space, the conversion from one mode of activity to another may involve physical movement of the polymerase on the DNA substrate and/or conformational changes within the polymerase or DNA. While structural studies have provided high resolution snapshots of DNA polymerases in specific functional states (Doublié et al., 1998; Kiefer et al., 1998; Johnson and Beese, 2004; Zahn et al., 2015; Huang et al., 2018; Jain et al., 2018; Jain et al., 2019; Hoitsma et al., 2020), much less is known about the conformational dynamics that underpin DNA polymerase activity.

Experiments performed at the single-molecule level can directly resolve conformational heterogeneity and provide kinetic information without the need to synchronize a population of molecules (Tinoco and Gonzalez, 2011; Monachino et al., 2017; Gruszka, 2021). With these capabilities, single-molecule studies have provided new mechanistic insights across a wide range of biological systems (Hilario and Kowalczykowski, 2010; Pljevaljčić et al., 2012; Hughes et al., 2014; McCluskey et al., 2014; Ordu et al., 2016; Banerjee et al., 2018; Okamoto et al., 2018; Jalihal et al., 2019; Kiss et al., 2020). Single-molecule studies of DNA polymerases and related replication enzymes have also been informative (Liu and Lou, 2017; Mueller et al., 2019; Bocanegra et al., 2021a; Bocanegra et al., 2021b). Observation of polymerase activity on single DNA templates under applied mechanical force have provided insights into the mechanochemistry of the elongation cycle (Maier et al., 2000; Wuite et al., 2000; Morin et al., 2015), strand displacement activity (Morin et al., 2012a; Morin et al., 2012b; Manosas et al., 2012) and the transition from polymerase activity to 3′-5′ exonuclease activity (Wuite et al., 2000; Ibarra et al., 2009; Hoekstra et al., 2017; Naufer et al., 2017). DNA polymerase activity has also been monitored at the single-molecule level using bioelectronic (Olsen et al., 2013) or nanopore (Cockroft et al., 2008; Lieberman et al., 2010; Olasagasti et al., 2010; Dahl et al., 2012; Lieberman et al., 2012; Lieberman et al., 2013) devices.

Fluorescence-based measurements provide additional insights by revealing conformational changes during polymerase activity. The spectroscopic phenomenon of Förster resonance energy transfer, which probes the distance between donor and acceptor fluorophores, is especially informative when monitored at the single-molecule level (Voith von Voithenberg and Lamb, 2018). Single-molecule Förster resonance energy transfer (smFRET) measurements can readily resolve different conformational states of a DNA polymerase, quantify their relative populations and the rate constants for exchange between them. Importantly, smFRET measurements can detect conformational transitions during a single encounter between a DNA polymerase and a DNA substrate, which can give insights into the mechanism of interconversion between different modes of polymerase activity.

The purpose of this review is to describe the smFRET methods that are used to probe the conformational dynamics of DNA polymerases. The review focuses on *E. coli* DNA polymerase I (Pol I), an A family polymerase, because this polymerase has been extensively studied by smFRET methods. Pol I is an informative model system because it contains template-directed 5′-3′ polymerase (*pol*), 3′-5′ exonuclease (*exo*) and 5′ nuclease (*5′ nuc*) activities in a single 928 aa polypeptide and does not require accessory proteins for proper function. The conformational dynamics underpinning all three activities of Pol I have been investigated by smFRET (Berezhna et al., 2012; Lamichhane et al., 2013; Pauszek et al., 2021). Related smFRET studies of other DNA polymerases are also described. The studies reviewed here provide new mechanistic insights into the biochemical functions of Pol I and highlight the novel information forthcoming from smFRET measurements of DNA polymerases.

### smFRET Measurements

For any FRET study, whether performed at the single-molecule or ensemble levels, it is necessary to covalently attach donor and acceptor fluorophores to the biomolecules of interest. In the case of DNA polymerases, one fluorophore can be attached to the DNA substrate and the other to the polymerase. Synthetic DNA oligonucleotides can be used as substrates for the polymerase and these can be readily labeled with fluorophores by incorporating amino-alkyl groups at the 3′ or 5′ ends or an internal base during oligonucleotide synthesis, followed by chemical reaction with succinimidyl ester fluorophore derivatives (Bailey et al., 2004). Site-specific fluorophore labeling of DNA polymerases can be achieved by introducing a single cysteine residue into the desired location, after removal of endogenous cysteines, followed by covalent attachment of maleimide fluorophore derivatives to the cysteine (Berezhna et al., 2012). Hence, to study a given polymerase using smFRET, it is necessary that the polymerase can be mutagenized at specific sites, expressed in reasonable yield (usually in bacteria) and purified to homogeneity. It is important to ensure that neither the protein mutations nor the presence of the fluorophores within the polymerase or DNA substrate perturb the enzymatic activity. An alternative approach is to introduce both donor and acceptor fluorophores into the polymerase. This requires creation of a double cysteine protein mutant, followed by covalent labeling with donor and acceptor (Santoso et al., 2010). Site-specific labeling can be achieved if the two cysteines have different reactivities towards maleimides (Santoso et al., 2010). Otherwise, the doubly labeled polymerase is a statistical mixture of the two possible labeling orientations.

One informative method for smFRET analysis is to attach DNA substrates to a quartz surface and to introduce a polymerase into the surrounding solution. A laser beam impinging on the surface at an angle of incidence beyond the critical angle for total internal reflection creates an evanescent field that penetrates ∼ 100–200 nm beyond the surface, allowing for selective excitation of surface-immobilized complexes. The laser is tuned to excite the donor and the emission from both donor and acceptor is recorded over time on a charge coupled device camera. The advantage of this approach is that hundreds of immobilized DNA molecules can be monitored in parallel and encounters between each DNA molecule and a polymerase from solution can be followed for extended periods of time (typically a few hundred seconds). A potential disadvantage is that the proximity of the surface perturbs the interaction between the polymerase and DNA. Accordingly, the quartz surface is generally passivated by a layer of polyethylene glycol (PEG) or similar polymers (Roy et al., 2008). A small fraction of the PEG molecules is biotinylated, enabling streptavidin or neutravidin to be captured on the surface. These multivalent proteins then serve to capture biotinylated DNA substrates on the passivated surface (Roy et al., 2008).

For every immobilized DNA molecule in the imaging field, the FRET efficiency at each time point, *E*(*t*), is calculated according to the following formula: *E*(*t*) = *I*_A_(*t*)/(*I*_A_(*t*) + *I*_D_(*t*)), where *I*_D_(*t*) and *I*_A_(*t*) are the corresponding donor and acceptor intensities (after any necessary instrumental corrections). A plot of *E*(*t*) versus *t*, known as a FRET trajectory, is constructed for each immobilized DNA. These trajectories can reveal abrupt changes in FRET efficiency as the polymerase switches between different DNA binding modes or different conformational states. Examples of FRET trajectories are shown in Figures 2, 5, 9, 10. Hundreds of individual FRET trajectories are then combined in the form of FRET efficiency histograms (shown in Figures 2, 5, 9, 10). These histograms reveal separate peaks (FRET states) corresponding to different DNA binding modes or polymerase conformations, and the areas enclosed by each peak reflect the equilibrium populations of each state. The FRET efficiency for each state is determined by the corresponding donor-acceptor distance, *R*_DA_, according to the formula: *E* = [1 + (*R*_DA_/*R*_0_)^6^]^−1^, where *R*_0_ is the Förster distance. The point of this analysis is not to determine *R*_DA_ itself, but rather to resolve different binding modes or polymerase conformations. To do so, the *R*_DA_ values for each state should be as different as possible, which is dependent on the donor and acceptor labeling positions and the underlying three-dimensional structure of the polymerase-DNA complex. While structural information can be helpful in selecting donor and acceptor labeling sites, these can also be identified by simple trial and error. Another informative statistical method of analysis is to compile two-dimensional plots of transition probability density (TPD) (McKinney et al., 2006). To do so, the final FRET efficiency is plotted versus the initial FRET efficiency for every transition observed in the entire set of FRET trajectories (typically thousands of transitions). These plots reveal prominent off-diagonal cross peaks that reflect how individual FRET states are interconnected. Examples of TPD plots are shown in Figures 5, 9, 10. Finally, to obtain kinetic information, each FRET trajectory is fitted using Hidden Markov modeling to provide the dwell times spent in one FRET state (state i) before transition to a different FRET state (state j) (McKinney et al., 2006). The dwell times from all trajectories are then compiled in the form of dwell time histograms. These histograms have an exponential shape and can be fitted with a single exponential function to quantify the rate constant for the state-to-state transition, *k*_ij_. An example of dwell time analysis is shown in Figure 6A. The depth of information available from these smFRET analyses is difficult to obtain using other biophysical or structural methods.

An alternative smFRET method is based on observation of freely diffusing molecules or complexes. In this approach, donor/acceptor-labeled polymerases or polymerase-DNA complexes are observed as they diffuse through the excitation volume of a tightly focused laser beam (tuned to excite the donor) (Santoso et al., 2010). Bursts of emission from both donor and acceptor are recorded on separate avalanche photodiode detectors. The advantage of this approach is that avalanche photodiodes have faster time resolution than charge-coupled device cameras, enabling observation of rapid conformational transitions, although the total observation period is limited by the transit time through the focal region. Another limitation is that molecules or complexes are observed one at a time, rather than in parallel, which reduces the throughput of data acquisition. Details of data acquisition and analysis, with specific reference to DNA polymerases, are described elsewhere (Hohlbein and Kapanidis, 2016). An elaboration of this method is to alternately excite the donor and acceptor with separate lasers, which provides information on both FRET efficiency and the stoichiometry of donor and acceptor labeling (Hohlbein and Kapanidis, 2016).

### Role of Polymerase Conformational Dynamics During Nucleotide Selection

DNA replication fidelity begins with the selection of the correct nucleotide substrate during template-directed polymerization of a nascent DNA strand. During each cycle, the polymerase selects the correct dNTP substrate from among the pool of all four possible substrates. While this selection is based on Watson-Crick pairing of the incoming nucleobase with the template base, the observed fidelity of correct nucleotide incorporation significantly exceeds that expected from thermodynamic differences between correct and incorrect base pairs (Kunkel and Bebenek, 2000; Kunkel, 2004), suggesting that the polymerase actively discriminates against incorrect pairings. This discrimination might be linked to conformational changes within the polymerase. X-ray crystallographic studies of DNA polymerases reveal an architecture akin to a human right hand, with thumb, fingers and palm domains (Ollis et al., 1985). In addition, structural studies have also revealed two major polymerase conformations, termed open and closed (Li et al., 1998). In the open conformation, the fingers are retracted away from the DNA substrate, leaving an open cavity that allows for binding of nucleotide substrates, while in the closed conformation the fingers close over the incoming nucleotide.

**FIGURE 1:**
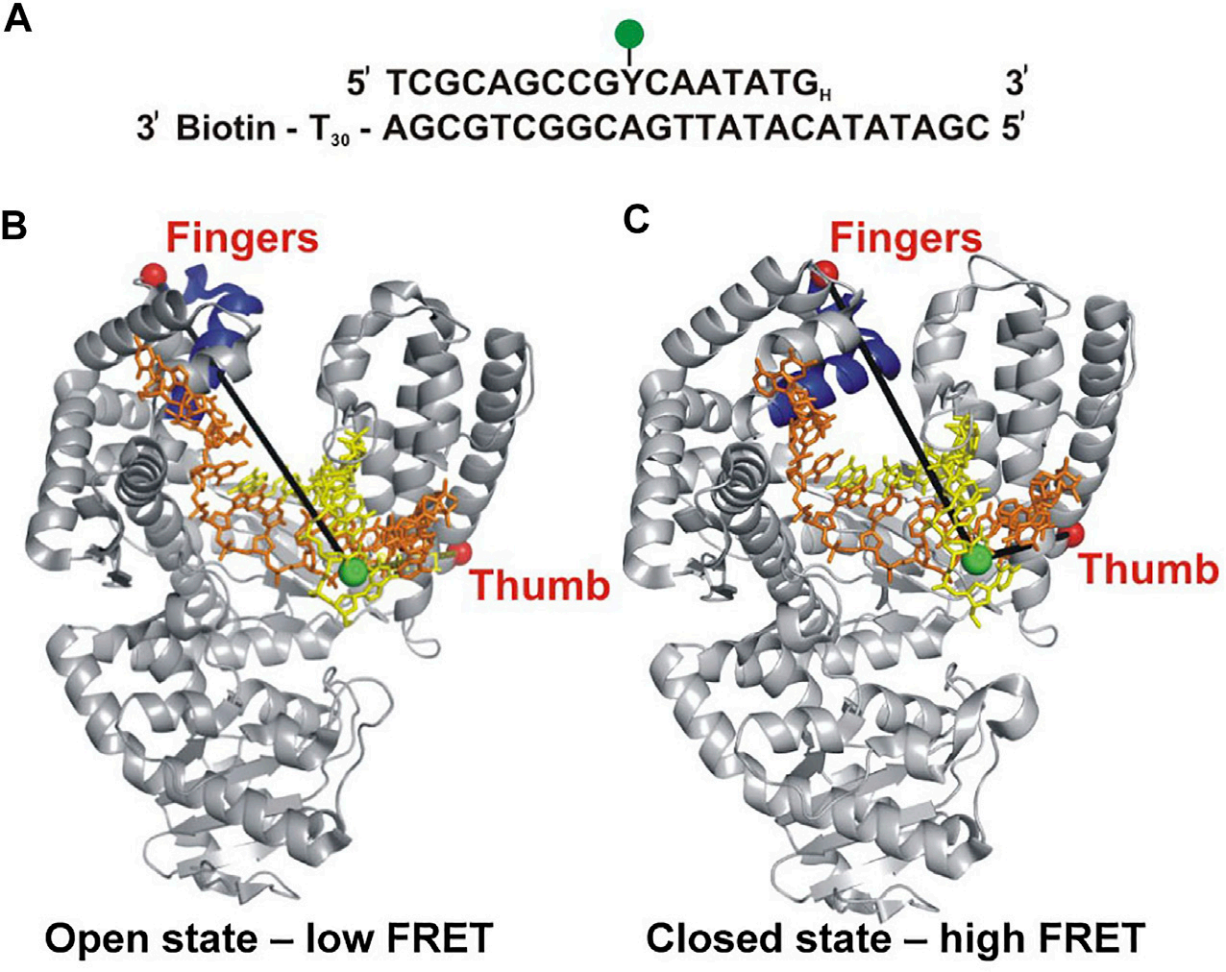
Labeling strategy used to probe the fingers-closure transition in KF. **(A)** Sequence of the primer/template. Y denotes the labeling site for A488 (green) and the H subscript at the 3′ end of the primer denotes a dideoxy modification. The biotin moiety on the template strand is used for surface attachment. **(B)** Crystal structure of the KF homolog *Bst* Pol I in open conformation (PDB code 1L3S). Primer strand is yellow and template strand is orange. **(C)** Crystal structure of *Bst* Pol I in closed conformation (PDB code 1LV5). In both **(B,C)**, the A488 donor is shown as a green sphere and the A594 acceptor attached to the fingers domain as a red sphere. The donor-acceptor distance (black lines) is shorter in the closed conformation than in the open conformation, leading to higher FRET efficiency. For some experiments (not described here), A594 was attached to the thumb domain. Reproduced from reference (Berezhna et al., 2012) with permission.

Berezhna et al. (Berezhna et al., 2012) designed a single-molecule spectroscopic system to detect open and closed conformations in solution and to investigate the possible linkage between nucleotide selection and the conformational dynamics of the polymerase. In this system, a defined oligonucleotide primer/template labeled with Alexa Fluor 488 (A488) at a specific base was immobilized on a quartz surface by biotin-neutravidin attachment (Figure 1A). The Klenow fragment (KF) of Pol I, present in solution, was labeled with Alexa Fluor 594 (A594) at a cysteine residue introduced into the fingers domain (at the tip of the O-helix) (Figure 1B). KF constitutes the main core of Pol I, but lacks the *5′ nuc* domain, allowing the nucleotide selection step to be decoupled from the *5′ nuc* activity of Pol I. The primer 3′ terminus contained a dideoxy modification, to block covalent incorporation of nucleotide substrates. Accordingly, this system was designed to probe conformational changes of KF that occur after nucleotide binding and precede the chemical step of covalent phosphodiester bond formation. The A488 and A594 fluorophores form a donor/acceptor (D/A) pair for measurement of Förster resonance energy transfer (FRET). The efficiency of FRET is strongly dependent on the D/A distance (Voith von Voithenberg and Lamb, 2018). Based on the expected shortening of the distance, the FRET efficiency should be higher in the closed conformation than in the open conformation (Figures 1B,C).

**FIGURE 2:**
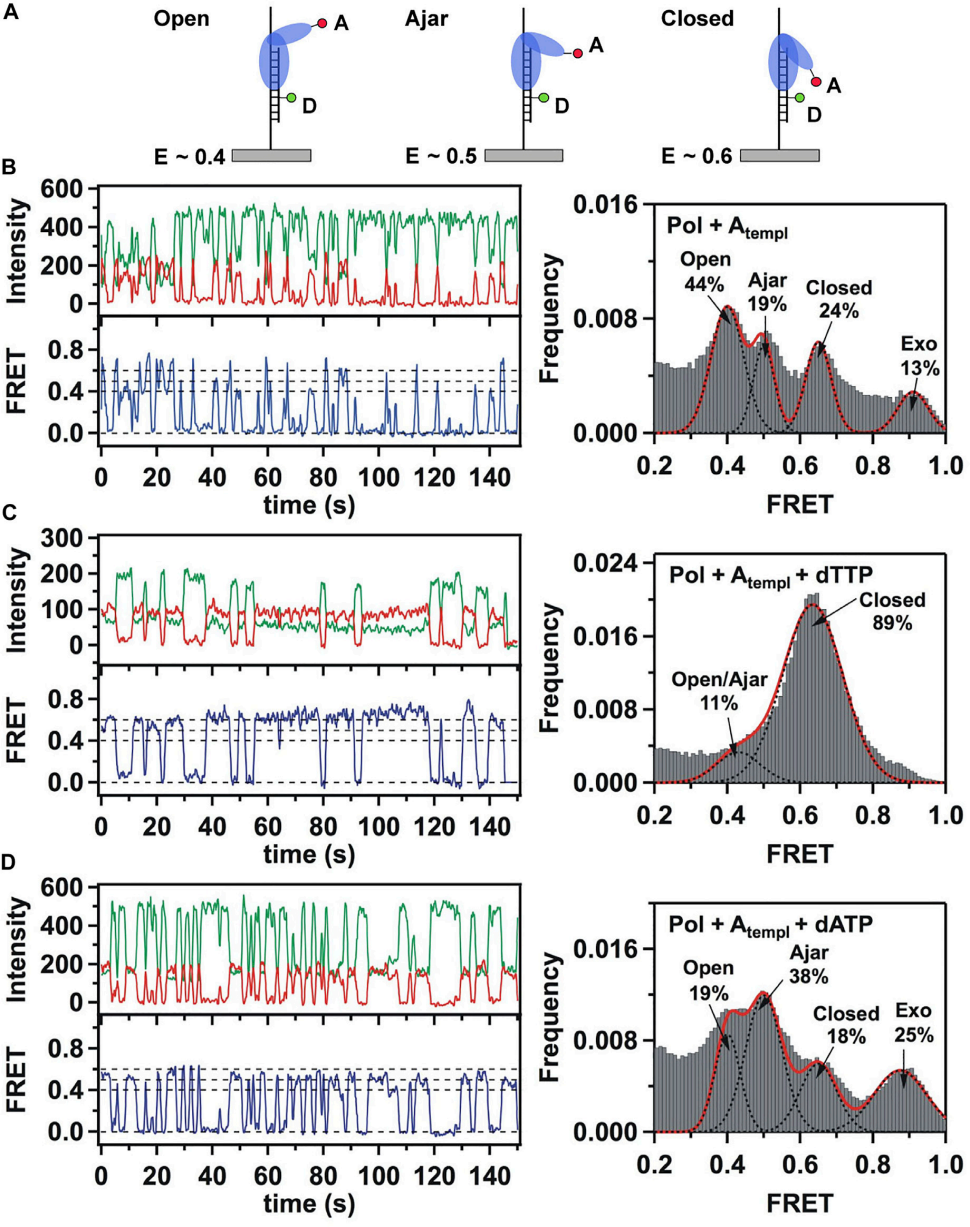
Single-molecule FRET analysis of the fingers closure transition in KF. **(A)** Schematic illustration of the three FRET states associated with different conformations of the fingers domain, with the observed FRET efficiencies indicated. **(B)** smFRET data in the absence of nucleotide substrates. Representative donor intensity (green), acceptor intensity (red) and corresponding FRET efficiency (blue) trajectories are shown on left. The perfect anticorrelation of the donor and acceptor intensity fluctuations is proof that FRET is occurring. The dashed horizontal lines superimposed on the FRET trajectory indicate the FRET efficiencies of the open (*E* = 0.4), ajar (*E* = 0.5) and closed (*E* = 0.6) states. Histogram of FRET efficiencies compiled from multiple traces and fit to four distinct states is shown at right. **(C)** In the presence of the correct dTTP substrate. **(D)** In the presence of the incorrect dATP substrate. Reproduced from reference (Berezhna et al., 2012) with permission.

**FIGURE 3:**
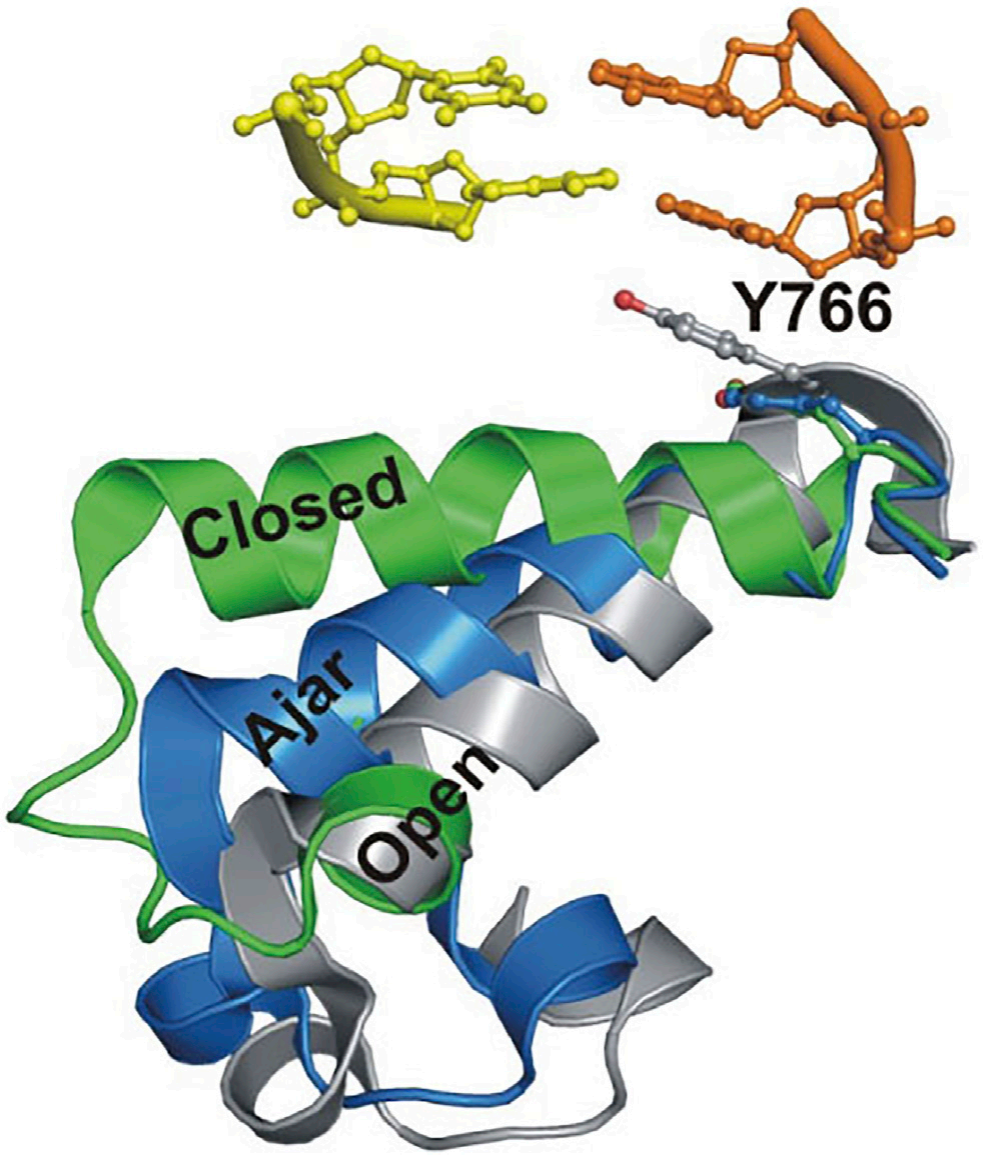
Overlay of crystal structures of *Bst* Pol I reveals three distinct conformations of the O-helix within the fingers domain. Adapted from reference (Berezhna et al., 2012) with permission.

This system was initially used to examine the behavior of the polymerase in the absence of any nucleotide substrates. The polymerase was highly labile under these conditions, transiently binding the immobilized DNA and sampling three distinct FRET states during the brief periods while bound (Figures 2A,B). While the low-FRET and high-FRET states were assigned to open and closed conformations, respectively (Figure 2A), the observation of an intermediate FRET state was initially unexpected. However, a crystal structure of *Bst* polymerase, a KF homolog, bound to DNA and a mismatched nucleotide revealed a third conformation of the fingers domain, intermediate between open and closed, termed “ajar” (Wu and Beese, 2011) (Figure 3). Accordingly, the intermediate FRET state was assigned to an ajar conformation of KF (Figure 2A). These observations revealed that KF is intrinsically dynamic in solution, sampling open, ajar and closed conformations, all of which were significantly populated (Figure 2B). The results also highlighted the ability of smFRET measurements to resolve conformational heterogeneity of a DNA polymerase.

Strikingly different behavior was observed in the presence of a correct nucleotide substrate (dTTP, complementary to the A templating base). Only the closed conformation was significantly populated, and the residence time of the polymerase on DNA was markedly prolonged, indicating more extensive contacts between DNA and the polymerase (Figure 2C). Presumably, the polymerase was poised to incorporate the nucleotide, which was blocked in this study by the dideoxy modification of the primer 3′ terminus. These results revealed that the presence of a correct nucleotide had a significant impact on the local conformational preference of the fingers domain and the global stability of the ternary enzyme-DNA-dNTP complex.

The polymerase was also examined in the presence of an incorrect nucleotide substrate (dATP, mismatched with respect to the A templating base). The polymerase dissociated rapidly after each encounter with the DNA substrate, reminiscent of the binary complex (Figure 2D). However, the population of the ajar state was markedly higher than in the binary complex, while the populations of the open and closed states were correspondingly reduced. Based on these observations, the ajar conformation was proposed to serve as a fidelity checkpoint to examine the identity of an incoming nucleotide substrate. In the presence of a correct nucleotide, the fingers domain rapidly proceeds to the closed conformation and the polymerase remains stably bound to DNA. However, in the presence of an incorrect nucleotide, the fingers domain remains in the ajar conformation and the polymerase rapidly dissociates from the DNA.

Another novel observation of the study by Berezhna et al. (Berezhna et al., 2012) was the appearance of a state with high FRET efficiency, clearly evident in the binary complex (Figure 2B). This state was assigned to a subpopulation of KF molecules engaging the immobilized primer/template *via* the *exo* site. This state vanished in the presence of a correct nucleotide (Figure 2C), indicating that the polymerase engaged the DNA exclusively *via* the *pol* site, as expected. Surprisingly, the high-FRET state was enhanced in the presence of the incorrect nucleotide substrate (Figure 2D), suggesting that the incorrect nucleotide promotes movement of the primer 3′ terminus from the *pol* site to the *exo* site. Shifting the primer terminus out of the *po*l site is an additional mechanism to suppress misincorporation of the incorrect nucleotide. This contribution to polymerase fidelity had not been recognized previously.

The fingers closure transition was also monitored using a complementary smFRET system in which KF was labeled with donor and acceptor in the fingers and thumb domains and freely diffusing polymerase molecules were detected as they passed through the focal volume of a laser (Santoso et al., 2010). This system enabled observation of the apo enzyme, as well as complexes with DNA and nucleotide substrates. Interestingly, the unliganded enzyme was observed to exchange rapidly between low-FRET and high-FRET states, assigned to open and closed conformations, showing that the fingers domain is an intrinsically mobile element of KF. Both open and closed conformations were observed in a binary KF-DNA complex, whereas the closed conformation was preferred in a ternary complex with DNA and a correct dNTP substrate. These observations mirror the results presented above (Figure 2). Hence, smFRET analyses of KF employing donor and acceptor within the DNA substrate and polymerase, described above, or both within the polymerase (Santoso et al., 2010; Hohlbein et al., 2013) yield consistent findings.

Doubly labeled KF was also examined in the presence of DNA and an incorrect dNTP (Santoso et al., 2010; Hohlbein et al., 2013). The low-FRET state showed a small shift to higher FRET efficiency, consistent with the formation of a partially closed conformation, presumably the ajar conformation. However, the open and ajar states were not resolved as separate peaks in the FRET histograms, as in Figure 2. It appears that the smFRET system described above (Figures 1, 2) is better able to resolve different conformations of the fingers domain. The diffusion-based smFRET system employing doubly labeled KF was also used to investigate the impact of polymerase mutations (with mutator phenotypes) on nucleotide recognition and the fingers closure transition (Hohlbein et al., 2013).

In a related study, doubly labeled KF was visualized as it interacted with immobilized (unlabeled) DNA primer/templates (Evans et al., 2015). In contrast to diffusion-based smFRET measurements, immobilization of DNA significantly extended the observation time window. This allowed measurement of the rate constants for closure and reopening of the fingers domain, both in the absence and presence of dNTP substrates. These observations provided new information on the pre-chemistry reaction steps in the nucleotide incorporation cycle of KF.

DNA polymerase B1 (PolB1) from *S. solfataricus*, a B family polymerase, has also been investigated using smFRET methods (Maxwell and Suo, 2013). The experimental strategy was similar to that described above for KF, utilizing a FRET donor attached to the primer strand of an immobilized DNA substrate and an acceptor attached to the fingers domain of the polymerase. This system revealed open and closed conformations of the fingers (but not an ajar conformation), as well as a subpopulation of DNA substrates bound at the *exo* site. Notably, the smFRET observations revealed transitions among these different states without dissociation from the DNA, as observed with KF (also see next section). Similar smFRET methods were used to monitor the fingers closure transition in DNA polymerase β (an X family polymerase) (Fijen et al., 2020) and Dpo4 polymerase (a Y family polymerase) (Raper and Suo, 2016).

### Role of Polymerase Conformational Dynamics During Proofreading

Despite the mechanisms that promote selection of correct nucleotide substrates and prevent misincorporation of incorrect nucleotides, mistakes are still sometimes made. Many DNA polymerases contain a 3′-5′ exonuclease activity to remove misincorporated nucleotides (proofreading) (Reha-Krantz, 2010; Bebenek and Ziuzia-Graczyk, 2018). Structural studies of A family (KF) (Freemont et al., 1988), B family (Berman et al., 2007) and C family (*E. coli* pol III) (Fernandez-Leiro et al., 2015; Fernandez-Leiro et al., 2017) polymerases have revealed that the active sites for *pol* activity and *exo* activity are spatially separated in distinct protein domains, indicating that mechanisms must exist to promote movement of a DNA substrate from the *pol* site to the *exo* site during proofreading.

**FIGURE 4:**
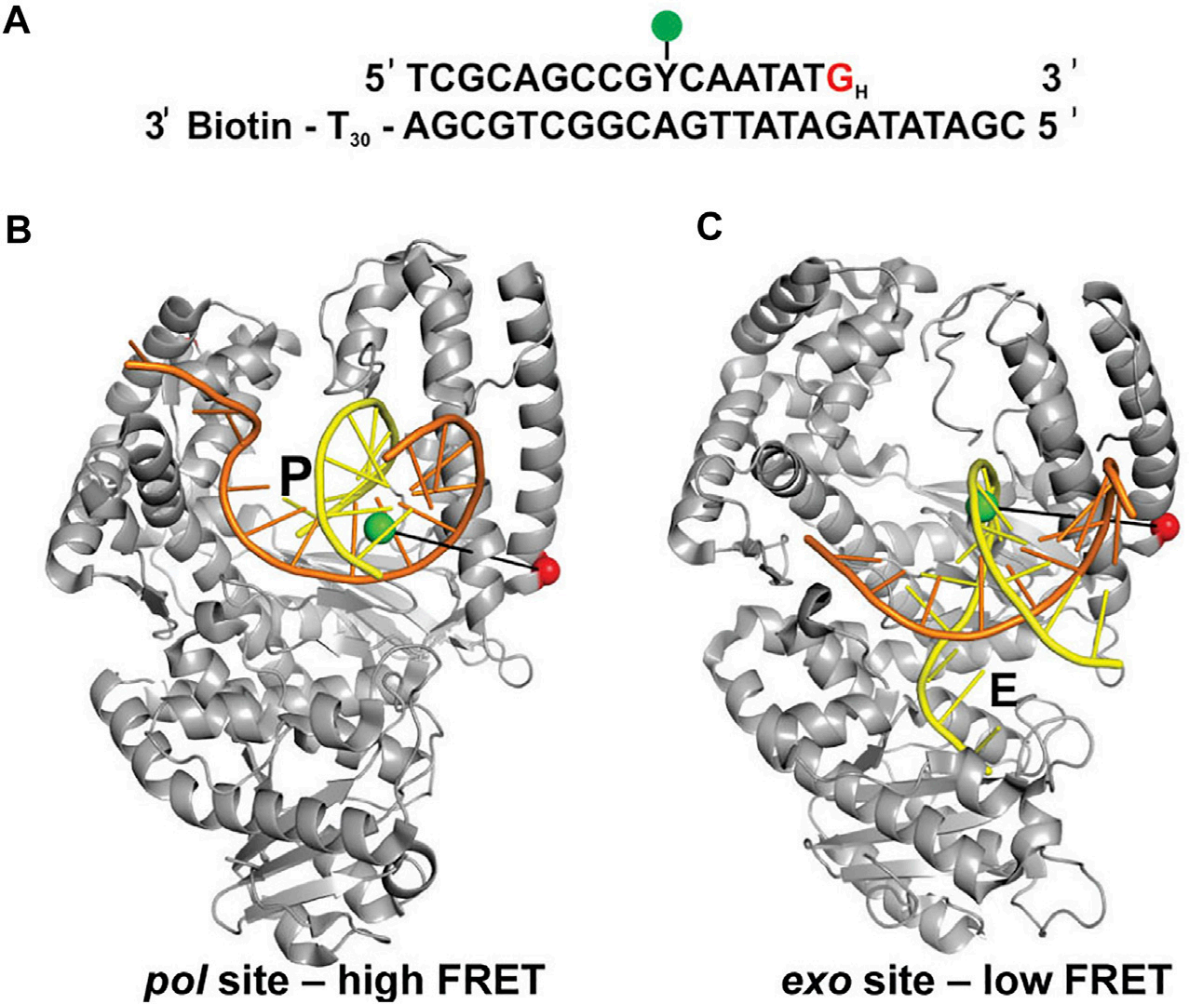
FRET labeling system designed to monitor switching of DNA between *pol* and *exo* sites of KF. **(A)** Sequence of primer/template containing terminal G:G mismatch. **(B)** Crystal structure of *Bst* Pol I with DNA at the *pol* site, P (PDB code 1L3S). Same strand colors as in Figures 1B,C. **(C)** Crystal structure of KF with DNA at the *exo* site, E (PDB code 1KLN). In **(B,C)**, the locations of the donor (green) and acceptor (red) are indicated. Different FRET efficiencies are expected for DNA bound to the *pol* site or the *exo* site. Adapted from reference (Lamichhane et al., 2013) with permission.

Lamichhane et al. (Lamichhane et al., 2013) developed a smFRET system to monitor switching of DNA between *pol* and *exo* sites in KF. A similar experimental strategy to the assay described in the previous section was employed, featuring an immobilized primer/template labeled with an A488 donor (Figure 4A). However, the A594 acceptor was attached to the thumb domain of the polymerase (*via* a K550C mutation), a rigid structural element that provides a static reference point to detect any physical movement relative to the DNA substrate (Figures 4B,C). Again, KF was used rather than full length Pol I, since the *pol* and *exo* sites are both contained within the KF portion. Different FRET efficiencies were expected for DNA substrates bound to the *pol* site or the *exo* site (Figures 4B,C). To promote occupancy of the *exo* site, the DNA substrate contained a G:G mismatch at the primer 3’ terminus (Figure 4A), mimicking the product of a misincorporation event.

**FIGURE 5:**
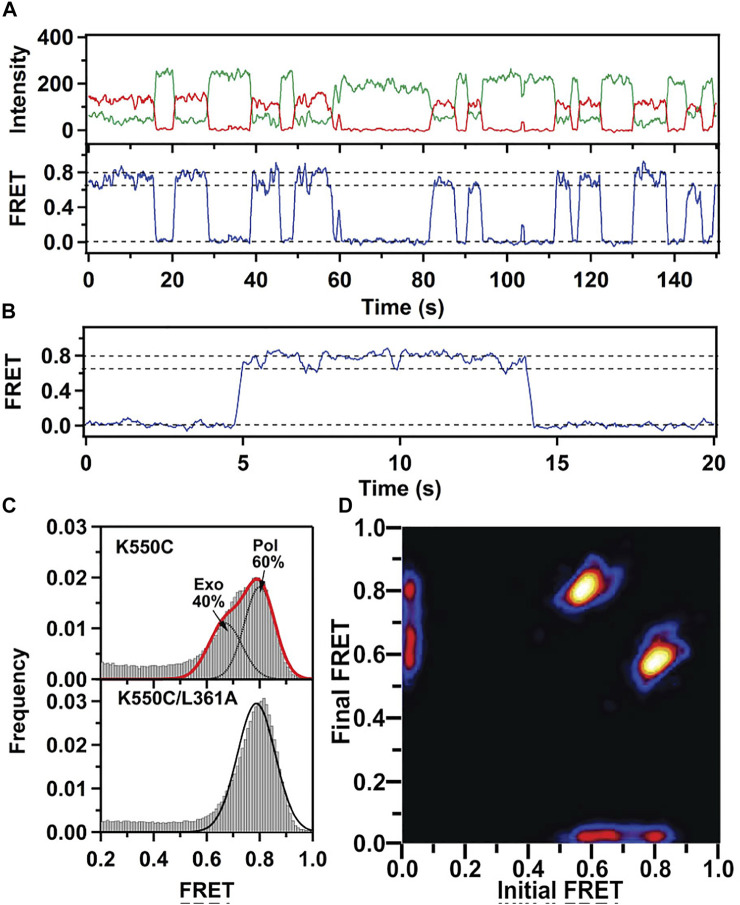
smFRET data for KF interacting with mismatched DNA. **(A)** Representative donor intensity (green), acceptor intensity (red) and FRET efficiency (blue) trajectories. **(B)** Expanded view of a single encounter between KF and DNA. **(C)** FRET efficiency histograms compiled from multiple traces. **(D)** Plot of transition probability density. Reproduced from reference (Lamichhane et al., 2013) with permission.

**FIGURE 6:**
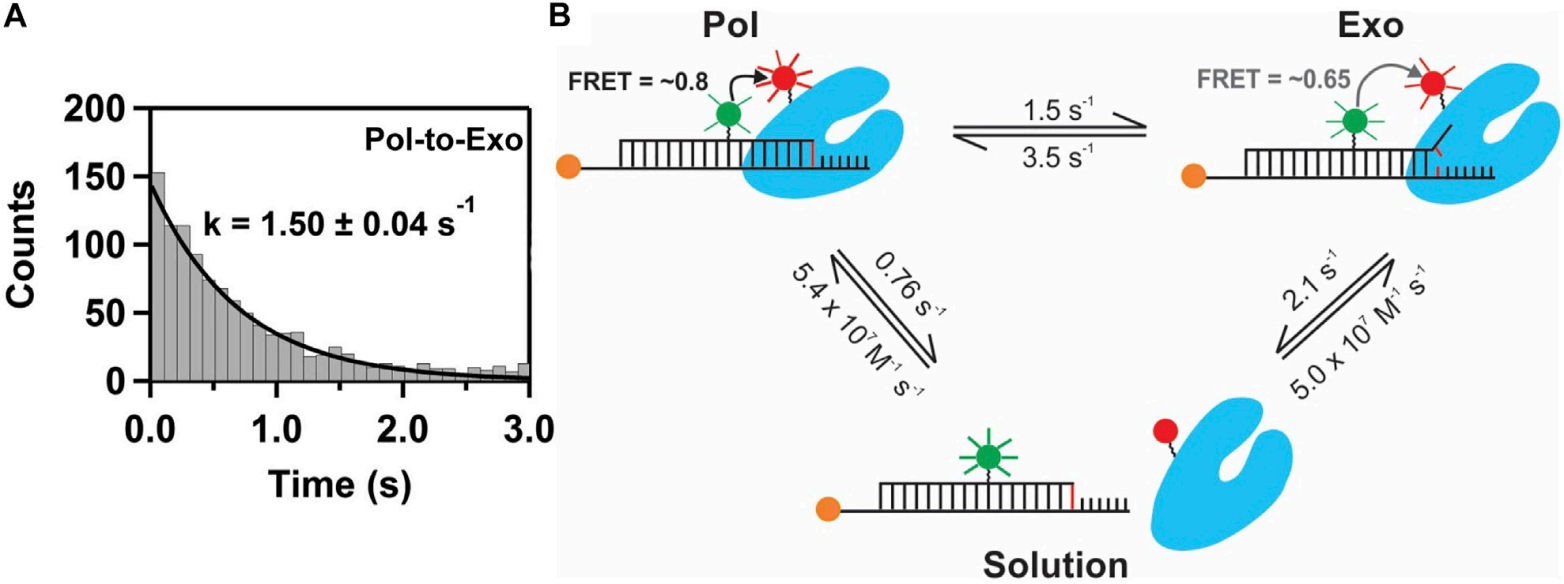
Pathways and rate constants for switching of DNA between *pol* and *exo* sites in KF. **(A)** Dwell time histogram for direct transitions from the *pol* site to the *exo* site. The smooth line is the best fit to a single exponential function, with the rate constant indicated. **(B)** Summary of the two observed transfer pathways. The intramolecular transfer pathway between bound complexes is shown at the top, together with the measured rate constants obtained from dwell time analysis of smFRET trajectories. The intermolecular pathway, involving dissociation from one site and rebinding from solution to the other site, is shown below, together with the respective rate constants.

This system exhibited two distinct FRET states, with FRET efficiencies ∼ 0.6 and ∼ 0.8, which were assigned to DNA occupying the *exo* site or *pol* site, respectively (Figure 5A). These assignments were confirmed using a L361A KF mutant, which is defective in binding DNA at the *exo* site (Figure 5C). Importantly, multiple transitions were observed between these FRET states during single encounters between KF and DNA (Figure 5B). These direct transitions were also manifested as prominent cross peaks in two-dimensional plots of transition probability density (TPD) (Figure 5D). These observations revealed that DNA substrates could transfer reversibly between the *pol* and *exo* sites while remaining associated with the polymerase. This intramolecular transfer mechanism had been inferred from earlier biochemical studies (Joyce, 1989) and was directly revealed by the smFRET results. Moreover, dwell time analysis was used to quantify the rate constants for intramolecular transfer of DNA between the two sites, in either direction (the dwell time histogram for transitions from the *pol* site to the *exo* site is shown in Figure 6A). The rate constants for the intramolecular transfer pathway are summarized in Figure 6B.

The smFRET results also revealed that KF bound to DNA *via* the *pol* site could dissociate into bulk solution and then rebind DNA *via* the *exo* site (and vice versa). The rate constants for this intermolecular transfer pathway were also determined from dwell time analysis (summarized in Figure 6B). During DNA replication *in vivo*, the polymerase is tethered to a replication fork *via* clamp proteins, which would inhibit dissociation of the complex. Intramolecular transfer of DNA is likely to be the relevant pathway during proofreading *in vivo*.

A recent computational study predicted the intramolecular transfer path between *pol* and *exo* sites in *E. coli* Pol III (Dodd et al., 2020). To test the model, the smFRET site switching assay originally developed for KF could be used to quantify how mutations of specific residues lying on the predicted transfer path in Pol III impact the switching kinetics.

### Role of Polymerase Conformational Dynamics During 5’ Nuclease Activity

**FIGURE 7:**
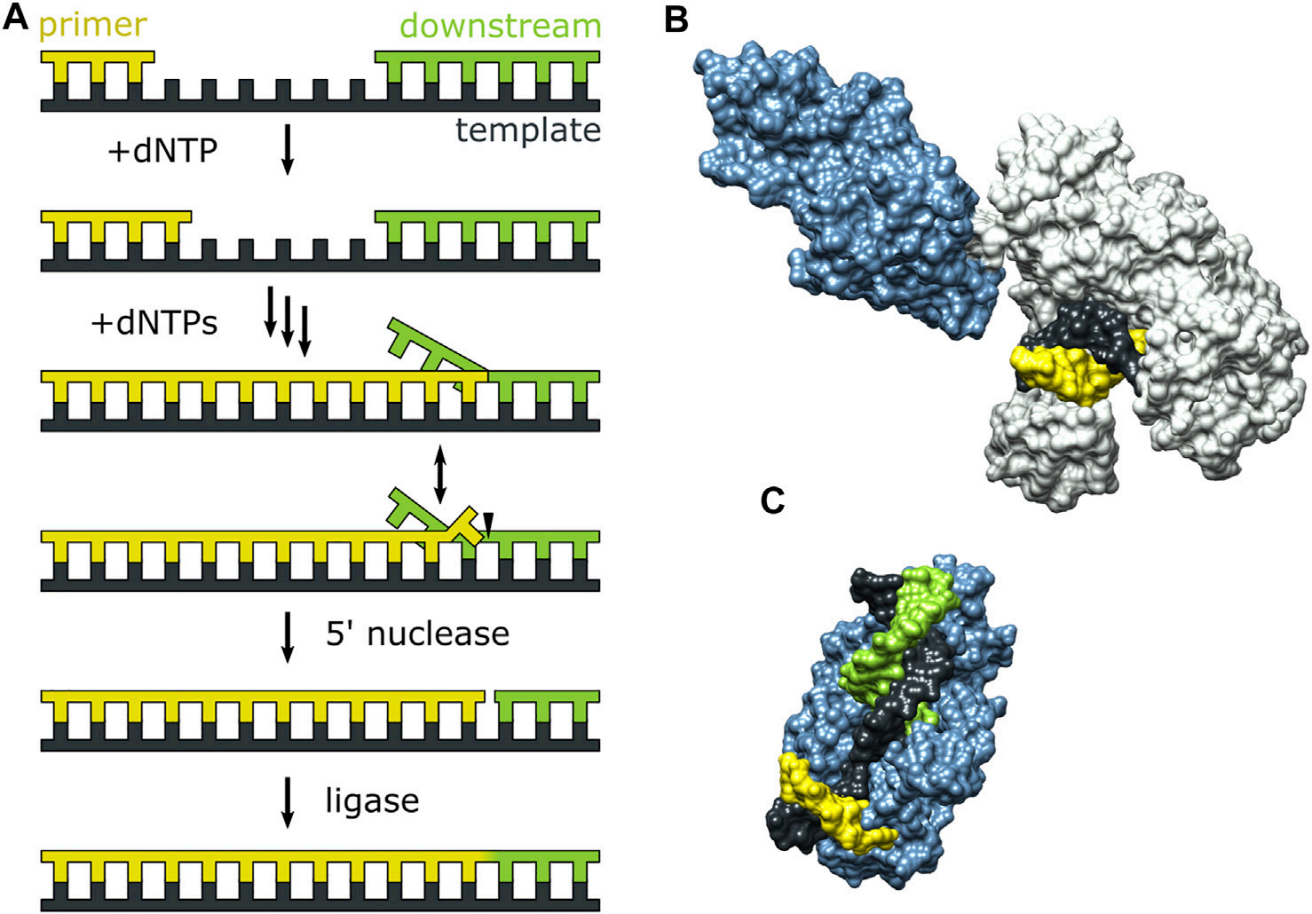
Activities of Pol I and three-dimensional structures of Pol I homologs. **(A)** Processes catalyzed by Pol I during lagging strand DNA synthesis and base excision repair. **(B)** Crystal structure of the Pol I homolog Taq polymerase with primer/template DNA at the *pol* site (PDB code 1TAU). The polymerase core is grey and the *5′ nuc* domain is blue. **(C)** Crystal structure of human FEN1 (homologous to the *5′ nuc* domain of Pol I) bound to a DNA substrate (PDB code 3Q8M). In **(B,C)**, the primer, template and downstream strands are colored as in **(A)**. Reproduced from reference (Pauszek et al., 2021) with permission.

Pol I can extend a primer strand in the presence of a downstream strand, resulting in displacement of the downstream strand and formation of a 5′ flap (Figure 7A). The flap is then cleaved by the 5′ nuclease (*5′ nuc*) activity of Pol I, leaving a nick that can subsequently be sealed by a DNA ligase. The processing steps depicted in Figure 7A are carried out by Pol I during lagging strand DNA synthesis (Balakrishnan and Bambara, 2013) and DNA base excision repair (Imai et al., 2007) in *E. coli*. The *5′ nuc* activity is contained in a separate domain that is connected to the main body of the enzyme (the KF portion) by a flexible 16 aa peptide linker (Figure 7B). Moreover, the *5′ nuc* domain is homologous to various structure-specific flap endonucleases (Figure 7C) (Harrington and Lieber, 1994).

**FIGURE 8:**
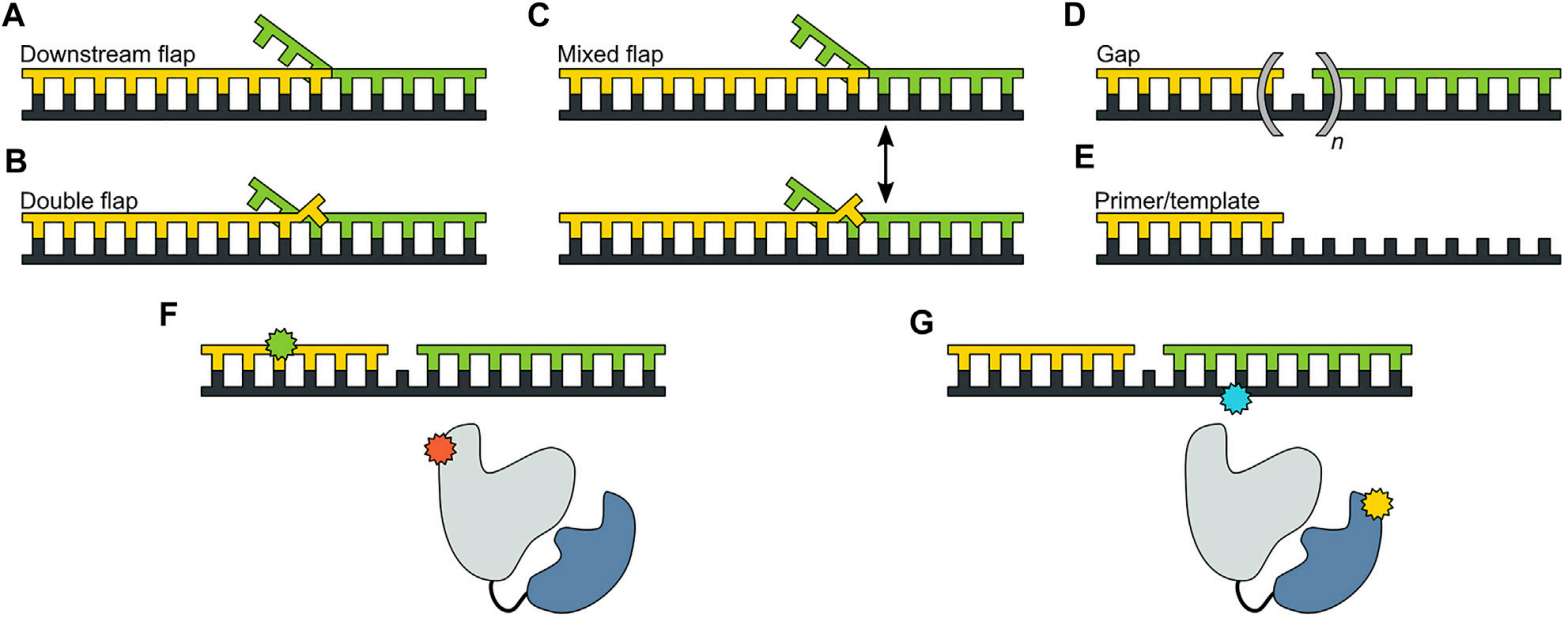
DNA substrates and FRET labeling strategies. **(A)** Substrate containing a 5′ flap on the downstream strand. **(B)** Substrate containing flaps on the primer and downstream strands (double-flap). **(C)** Substrate that exists as a mixture of downstream flap and double-flap forms. **(D)** Substrates containing gaps of various sizes. **(E)** Primer/template substrate, lacking a downstream strand entirely. **(F)** First FRET labeling scheme. Donor is green and acceptor is red. **(G)** Second FRET labeling scheme. Donor is yellow and acceptor is blue. Reproduced from reference (Pauszek et al., 2021) with permission.

The *pol* and *5′ nuc* activities of Pol I must be carefully coordinated to ensure that the resulting DNA product contains a nick rather than an extended gap or overhanging 5′ strand. However, the physical basis for this coordination is not well understood. Pauszek et al. (Pauszek et al., 2021) designed a single-molecule FRET system to monitor the transition from *pol* activity to *5′ nuc* activity in Pol I. A variety of DNA substrates (Figures 8A–E) and two complementary labeling schemes were employed (Figures 8F,G). The first scheme was identical to that used to monitor *pol* to *exo* site switching in KF (same donor and acceptor sites), except that full length Pol I was employed (Figure 8F). This system was designed to monitor any movement of the DNA substrate relative to the enzyme core. The second FRET scheme employed an A488 donor attached to the *5′ nuc* domain of Pol I and an A594 acceptor attached to the template strand downstream of the primer 3′ terminus (Figure 8G). This scheme was designed to probe the proximity of the *5’ nuc* domain to the downstream DNA.

**FIGURE 9:**
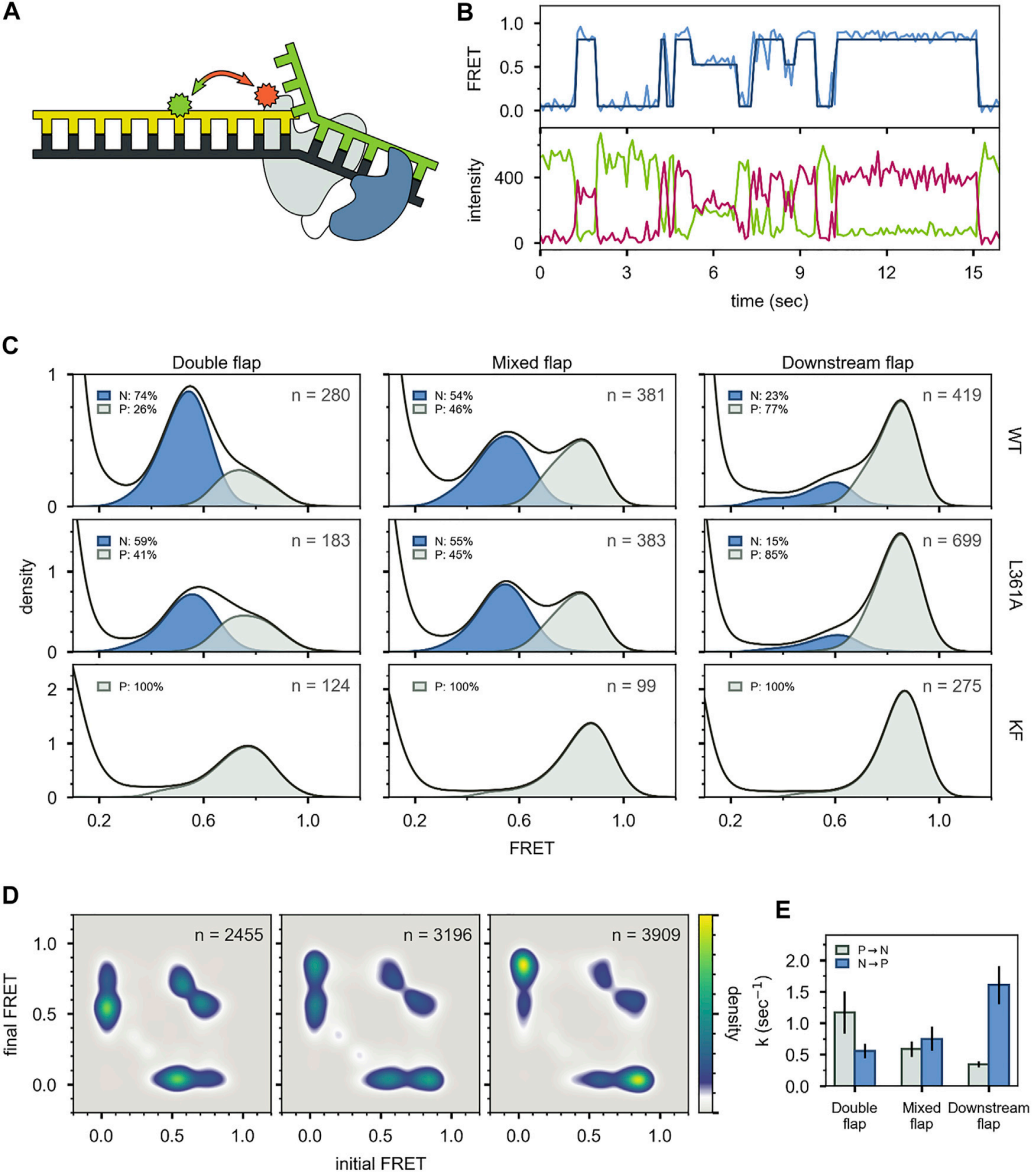
smFRET data for Pol I—DNA complexes obtained using labeling. **(A)** Locations of donor (green) and acceptor (red). Polymerase core is grey and *5′ nuc* domain is blue. **(B)** Donor intensity (green), acceptor intensity (red) and FRET efficiency (blue) trajectories are shown. The black line is a fit from Hidden Markov modeling. **(C)** FRET efficiency histograms compiled from n trajectories, separated into states P and N. **(D)** Transition probability density plots, compiled from n transitions. From left to right: double flap DNA, mixed flap DNA and downstream flap DNA. **(E)** Rate constants for intramolecular transitions between states P and N. Reproduced from reference (Pauszek et al., 2021) with permission.

For the first labeling scheme (Figure 9A), Pol I exhibited two distinct FRET states, ∼ 0.6 and ∼ 0.8 efficiency, when interacting with any of the flap-containing DNA substrates (Figures 9B,C). The population of the 0.6 FRET state was not responsive, or only weakly responsive, to a L361A mutation in Pol I (Figure 9C), indicating that this state was not associated with binding of DNA to the *exo* site. In contrast, the 0.6 FRET state was not observed for any of the flap-containing DNAs interacting with KF (Figure 9C), which lacks the *5′ nuc* domain entirely. Based on these results, the 0.8 and 0.6 FRET states were assigned to DNA engaging the *pol* site (state P) or the *5′ nuc* site (state N), respectively. State N was most highly populated with the double-flap DNA (Figure 9C), which is the natural substrate for *5′ nuc* activity (blocked here by a D116A mutation).

**FIGURE 10:**
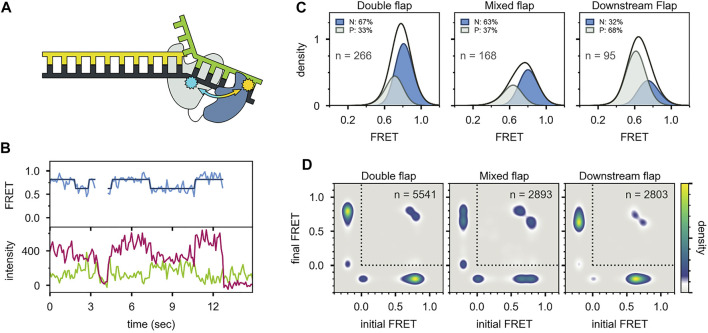
smFRET data for Pol I—DNA complexes obtained using the second labeling scheme. **(A)** Locations of donor (yellow) and acceptor (blue). The polymerase core is grey and *5′ nuc* domain is blue. **(B)** Donor intensity (green), acceptor intensity (red) and FRET efficiency (blue) trajectories are shown. The black line is a fit from Hidden Markov modeling. **(C)** FRET efficiency histograms compiled from n trajectories, separated into states P and N. **(D)** Transition probability density plots, compiled from n transitions. Reproduced from reference (Pauszek et al., 2021) with permission.

**FIGURE 11:**
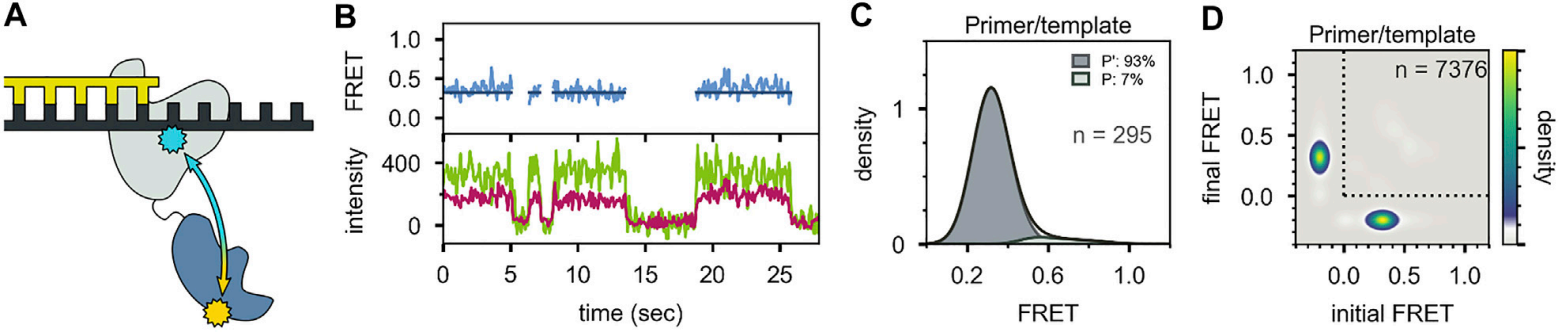
Probing the location of the *5′ nuc* domain in the absence of a downstream DNA strand. **(A)** Schematic representation of donor (yellow) and acceptor (blue) sites. **(B)** Donor intensity (green), acceptor intensity (red) and FRET efficiency (blue) traces are shown. The black line is a fit from Hidden Markov modeling. **(C)** FRET efficiency histogram compiled from n trajectories, separated into states P′ and P. **(D)** Transition probability density plot, compiled from n transitions. Reproduced from reference (Pauszek et al., 2021) with permission.

Two distinct FRET states were also observed with the second labeling scheme (Figures 10A-C), which probes the proximity of the *5′ nuc* domain to the downstream DNA. The high-FRET and mid-FRET states were assigned to states N and P, respectively, based on the correspondence in the species populations observed with the two labeling schemes. Hence, the two FRET schemes yield a consistent description of the conformational states populated with flap-containing DNA substrates. Moreover, the results with the second labeling scheme confirmed that the *5′ nuc* domain was in close physical proximity to the downstream DNA in state N. Notably, only a single low-FRET state was observed for a primer/template substrate, indicating that the *5′ nuc* domain was extended away from the DNA (Figures 11A-D). Thus, the presence of a downstream strand is necessary to engage the *5′ nuc* domain. This low-FRET state was designated P′ (see below).

For both FRET systems, direct transitions were observed between states P and N during single encounters between Pol I and the flap-containing DNAs (Figure 9B, Figure 10B). Similarly, prominent cross-peaks were observed in two-dimensional TPD plots (Figure 9D, Figure 10D). These observations establish that DNA substrates initially engaging the *pol* site can switch to the *5′ nuc* site while remaining associated with Pol I (and vice versa). Hence, transfer of DNA from the *pol* site to the *5′ nuc* site is governed by an intramolecular pathway, as observed for DNA switching between *pol* and *exo* sites. Rate constants for site switching were determined from dwell-time analysis (Figure 9E). The double-flap DNA substrate exhibited the fastest transfer from the *pol* site to the *5′ nuc* site and the slowest return to the *pol* site, accounting for the high population of state N (Figure 9C).

**FIGURE 12:**
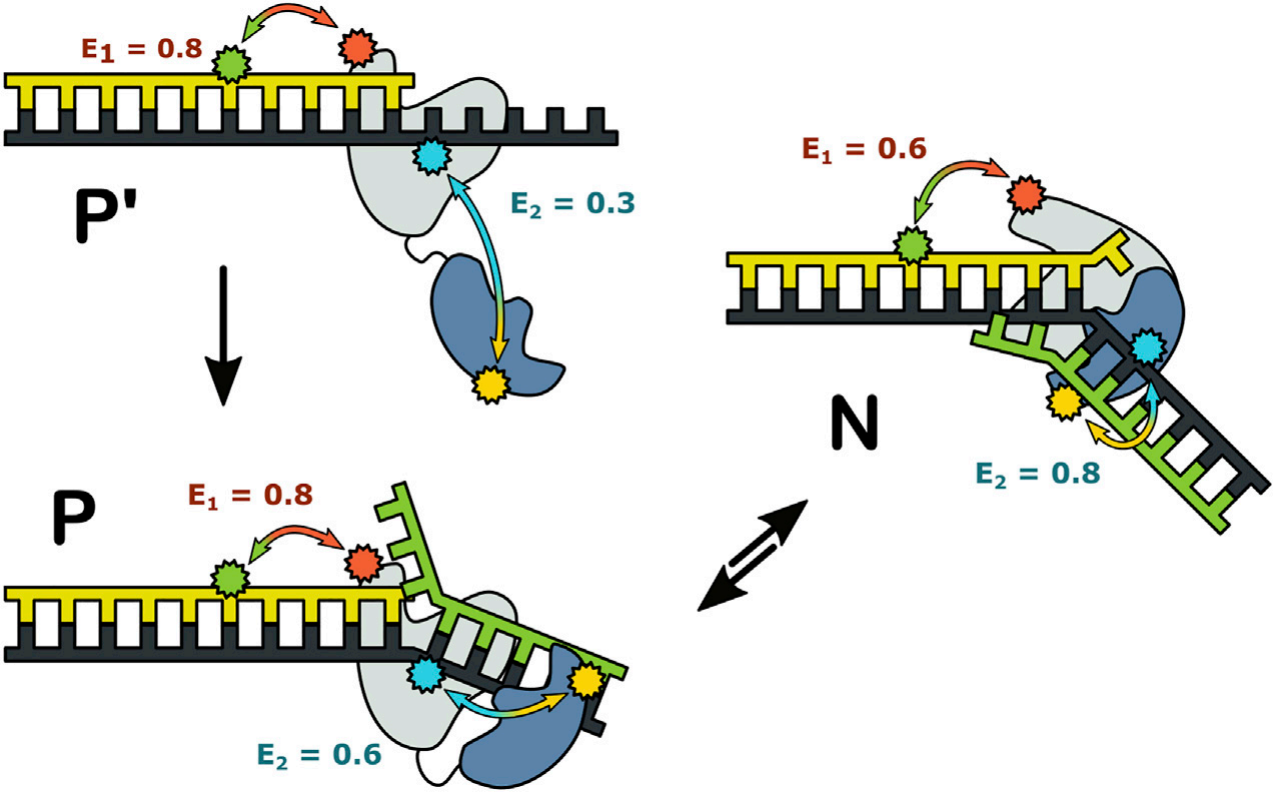
Possible configurations of Pol I—DNA complexes. The FRET efficiencies measured with labeling are denoted E_1_ and E_2_, respectively. Reproduced from reference (Pauszek et al., 2021) with permission.

Together, these observations revealed that three distinct complexes can form during encounters between Pol I and DNA substrates (Figure 12). State P′ is formed when the primer 3′ terminus is remote from a downstream strand: the primer terminus is positioned in the *pol* site and the *5′ nuc* domain is extended away from the DNA. State P is formed as the primer 3′ terminus approaches a downstream strand: the primer terminus is still located in the *pol* site, but the *5′ nuc* domain is in proximity to the downstream DNA. State N is formed after the primer 3′ terminus has shifted out of the *pol* site and the *5′ nuc* domain is poised to cleave the downstream strand. Importantly, the smFRET results showed that states P and N can reversibly exchange without dissociation, explaining how the *pol* and *5’ nuc* modes of activity are physically coordinated in Pol I.

### Other Applications of smFRET to DNA Polymerases

While the central focus of this review has been on the conformational dynamics underpinning the DNA replication fidelity of Pol I, smFRET methods have also been used to examine other aspects of DNA polymerase function. In an early application, smFRET was used to monitor the movement of KF on a DNA template during DNA synthesis, enabling measurement of primer elongation with single base-pair resolution (Christian et al., 2009). This approach was also used to elucidate the impact of bulky benzopyrene adducts on translesion DNA synthesis by the Y family polymerase Dpo4 (Liyanage et al., 2017).

Craggs et al. studied the structure-specific recognition of gapped DNA substrates by KF (Craggs et al., 2019). A large set of DNA substrates containing donors and acceptors at various sites were examined by smFRET, both in the presence and absence of KF. Using a docking approach based on the resulting network of D/A distances, a solution structure of the gapped DNA bound by KF was established, revealing a sharp bend in the DNA. This comprehensive study exemplified how smFRET data can be combined with structural modeling methods to provide a detailed structural description of an intrinsically dynamic macromolecular complex. Interestingly, the results also showed that the DNA alone could adopt a similar bent conformation, supporting a model wherein KF recognized a pre-bent DNA conformation.

## Concluding Remarks

The smFRET studies of Pol I, reviewed above, highlight the intrinsically dynamic character of the enzyme. Each of the three biochemical activities is linked to conformational dynamics of the enzyme-DNA complex. Moreover, the results reveal the different physical mechanisms employed in each case: 1) nucleotide selection is coupled to movement of the fingers domain, 2) proofreading involves physical movement of the DNA substrate between separated *pol* and *exo* sites, and 3) the *5′ nuc* activity requires both movement of DNA and a large conformational change of the polymerase (docking of the *5’ nuc* domain with the downstream DNA).

The smFRET methods reviewed here can readily resolve different binding modes of a polymerase-DNA complex, quantify their relative populations and the rates of exchange between them. The results also establish whether a polymerase switches from one mode of activity to another during a single encounter between the polymerase and DNA substrate (intramolecular events) or after dissociation and rebinding (intermolecular events). With the ability to label both the DNA substrate and the enzyme at specific locations with donor and acceptor probes, smFRET provides a general tool to probe the conformational dynamics of DNA polymerases in a region-specific manner. Further highlighting the versatility of the method, smFRET can also be used to monitor polymerase-mediated primer extension or to establish structural features of polymerase-DNA complexes. With these wide-ranging capabilities, smFRET is emerging as a powerful adjunct to traditional three-dimensional structural analyses of DNA polymerases.

The smFRET studies of Pol I performed to date were conducted under equilibrium conditions, in which the various enzymatic activities were blocked by enzyme mutations or modifications to the DNA substrate. Future smFRET studies employing fully active Pol I and extendable DNA substrates should reveal the sequence of polymerase conformational changes and DNA movements during each of the DNA processing steps performed by Pol I.

While this review has focused on *E. coli* Pol I, DNA polymerases from other organisms also possess multiple enzymatic activities that must be carefully coordinated to ensure efficient and accurate DNA replication and repair. In many polymerases, including those from eukaryotes, the various enzymatic activities are contained in distinct protein subunits within a multi-protein holoenzyme complex (McHenry, 2011; Burgers and Kunkel, 2017; Raia et al., 2019). The resulting spatial separation of the various active sites poses the same challenge facing Pol I: how to regulate the movement of a DNA substrate between physically remote active sites ? The mechanisms employed by Pol I to coordinate it is three activities, involving intramolecular shuttling of DNA between spatially separated active sites, combined with enzyme conformational changes, are likely employed across the DNA polymerase family. Accordingly, the smFRET methods reviewed here provide general tools to understand the physical basis of functional coordination in multi-functional DNA polymerases.
